# Early pregnancy exposure to endocrine disrupting chemical mixtures are associated with inflammatory changes in maternal and neonatal circulation

**DOI:** 10.1038/s41598-019-41134-z

**Published:** 2019-04-01

**Authors:** Angela S. Kelley, Margaret Banker, Jaclyn M. Goodrich, Dana C. Dolinoy, Charles Burant, Steven E. Domino, Yolanda R. Smith, Peter X. K. Song, Vasantha Padmanabhan

**Affiliations:** 1 Department of Obstetrics and Gynecology, University of Michigan, L4001 Women’s Hospital, 1500 East Medical Center Drive, Ann Arbor, Michigan 48109 USA; 2 Department of Biostatistics, University of Michigan School of Public Health, 1415 Washington Heights, Ann Arbor, Michigan 48109 USA; 3 Department of Environmental Health Sciences, University of Michigan School of Public Health, 1415 Washington Heights, Ann Arbor, Michigan 48109 USA; 4 Department of Nutritional Sciences, University of Michigan School of Public Health, 1415 Washington Heights, Ann Arbor, Michigan 48109 USA; 5 Department of Internal Medicine, University of Michigan, 24 Frank Lloyd Wright Drive, Ann Arbor, Michigan 48105 USA; 6 Department of Pediatrics, University of Michigan, 7510 MSRB 1, 1500 W. Medical Center Dr., Ann Arbor, MI 48109 USA

## Abstract

Endocrine disrupting chemicals (EDCs) are ubiquitous, and pregnancy is a sensitive window for toxicant exposure. EDCs may disrupt the maternal immune system, which may lead to poor pregnancy outcomes. Most studies investigate single EDCs, even though “real life” exposures do not occur in isolation. We tested the hypothesis that uniquely weighted mixtures of early pregnancy exposures are associated with distinct changes in the maternal and neonatal inflammasome. First trimester urine samples were tested for 12 phthalates, 12 phenols, and 17 metals in 56 women. Twelve cytokines were measured in first trimester and term maternal plasma, and in cord blood after delivery. Spearman correlations and linear regression were used to relate individual exposures with inflammatory cytokines. Linear regression was used to relate cytokine levels with gestational age and birth weight. Principal component analysis was used to assess the effect of weighted EDC mixtures on maternal and neonatal inflammation. Our results demonstrated that maternal and cord blood cytokines were differentially associated with (1) individual EDCs and (2) EDC mixtures. Several individual cytokines were positively associated with gestational age and birth weight. These observed associations between EDC mixtures and the pregnancy inflammasome may have clinical and public health implications for women of childbearing age.

## Introduction

Environmental toxicants, which include endocrine disrupting chemicals (EDC) such as phenols, phthalates, metals, and organochlorines, are globally ubiquitous and represent an area of major public health concern^1,2^. Data from the National Health and Nutrition Examination Survey (NHANES) show that the vast majority of US adults have evidence of exposure to phthalates and phenols, such as bisphenol A (BPA) and polyfluoroalkyl chemicals, as measured in urine samples^3–6^. Children also demonstrate exposure to a wide variety of environmental chemicals^7,8^. EDC exposure may occur on a daily basis via packaged foods, plastics, cosmetics, and pharmaceuticals, and industrial applications may lead to widespread environmental exposure to heavy metals^2,9,10^.

EDC exposures are of particular significance to pregnant women, as fetal development is sensitive to maternal nutritional, chemical, and environmental stressors. In utero exposures may compromise early developmental processes and predispose the fetus to adverse health risks later in life, based on the Developmental Origins of Health and Disease (DOHaD) hypothesis^11–13^. Epidemiologic studies have demonstrated that EDC exposures are nearly universal in pregnant women^14–18^. Exposure to EDC classes including phenols, phthalates, parabens, flame retardants, and heavy metals may occur in pregnancy by way of personal hygiene products, cosmetics, household cleaning products, the use of electronic devices, and consumption of animal, plant, or processed foods^19,20^.

Pregnancy is a clinically relevant susceptibility period for investigating EDC exposures, due to the growing body of evidence that EDCs modulate physiologic processes in both exposed individuals and their offspring^2^. Epidemiologic studies have also demonstrated that prenatal exposure to BPA, phthalates, and polyfluoroalkyl chemicals may be associated with fetal growth restriction or small for gestational age babies^21–27^, both of which are risk factors for adult onset disease^28–30^. However, the literature is mixed, with some studies demonstrating no effect of EDC exposure on birth outcomes or anthropometric measures^31,32^.

Biologically, EDCs may mimic or interfere with estrogenic, androgenic, glucocorticoid, thyroid, and insulin signaling pathways, leading to a variety of downstream, tissue-specific effects^33,34^. EDCs may exert physiologic effects at low doses, and modulation of hormonal pathways occurs in a non-monotonic manner for substances such as BPA, dioxin, lead, and cadmium^33,35,36^. While most EDCs have been studied in isolation, there is increasing interest in the cumulative impact of EDC exposures, as humans are exposed to a multitude of EDCs at varying doses which may have additive, synergistic, or negative biologic effects^37–39^.

EDC exposures in pregnancy have been associated with changes in the gestational endocrine milieu, including altered levels of sex steroids^40,41^. Further, there is evidence that EDCs such as BPA and polycyclic aromatic hydrocarbons, and metals such as arsenic, cadmium, lead, and mercury, may modulate the immune system and alter inflammatory cytokine milieu to favor a pro-inflammatory state^42–44^. Meanwhile, findings from other pregnancy cohorts have demonstrated that urinary paraben and phenol measures may affect circulating markers of inflammation including IL-6, IL-10, CRP, and TNF-α^45,46^. Importantly, aberrant maternal inflammatory pathways have been linked to adverse pregnancy outcomes including pregnancy loss, preterm labor, preeclampsia, and fetal growth restriction^47–50^. Epidemiologic and animal studies indicate that such alterations induced in the maternal environment by EDCs may alter the developmental trajectory of the developing fetus via epigenetic modifications^51^.

The purpose of our pilot study is to use the Michigan Mother-Infant Pairs (MMIP) birth cohort to assess the impact of phenols, phthalates, and metals, both individually and in combination, on the maternal and neonatal inflammasome. Using maternal urinary measures of environmental toxicants during the first trimester, a sensitive period in fetal development, we correlated exposures to plasma levels of inflammatory cytokines in both maternal (at two time points) and neonatal umbilical cord blood. Exposures and inflammatory cytokine levels were then correlated with birth outcomes, namely infant birth weight and gestational age at delivery.

## Methods

### Subject recruitment

The MMIP project is an ongoing birth cohort study (2010-present) at the University of Michigan (UM). Pregnant women are recruited into MMIP at their first prenatal appointment between 8 and 14 weeks of gestation. Those eligible for MMIP participation are between 18 and 42 years old, have a naturally conceived singleton pregnancy, and intend to deliver at UM. Pregnancy dating is determined by the obstetric provider, either based on last menstrual period or ultrasound. As of October 2018, 289 women in the MMIP cohort have delivered. Other studies involving subsets of MMIP participants have been published previously^21,52,53^.

A subset of fifty-six women, recruited between November 2012 and May 2015, were selected for the specific analyses described here. All 56 women met additional inclusion criteria as follows: 1. had available biospecimens from mother and infant, at all relevant time points, and 2. had provided complete demographic and health information at their initial study visit. Study procedures were approved by the UM Institutional Review Board, and all participants provided written informed consent. All research was performed in accordance with relevant guidelines and regulations.

### Sample collection

Spot urine and venous blood samples were collected from participants at their first prenatal appointment between 8 and 14 weeks of gestation, and upon arrival to the hospital for delivery, prior to IV placement. Urine samples were collected in polypropylene containers before being transferred to glass vials. All samples were stored at −80 degrees Celsius until analysis.

### Exposure assessments: EDCs, metals and metalloids

In total, 41 exposure measures were quantified per subject. These 41 exposures comprise a wide range of commonly encountered environmental toxicants and were chosen due to their heterogeneity.

Details on phthalate and phenol exposure assessment have been previously published by Montrose *et al*., 2018^53^. Briefly, using maternal urine samples collected between 8 and 14 weeks’ gestation, 12 phthalate metabolites and 12 phenol metabolites were quantified via isotope dilution liquid chromatography and tandem mass spectrometry.

Phthalate analytes included metabolites of diethylhexyl phthalates (DEHP): mono (2-ethyl-5-carboxylpentyl) phthalate (MECPP), mono (2-ethyl-5-hydroxyhexyl) phthalate (MEHHP), mono (2-ethylhexyl) phthalate (MEHP), and mono (2-ethyl-5-oxohexyl) phthalate (MEOHP); and dibutyl phthalates (DBP): mono-isobutyl phthalate (MIBP) and mono n-butyl phthalate (MnBP). Concentrations of mono-benzyl phthalate (MBzP), mono-carboxy isononyl phthalate (mCINP), mono (3-carboxypropyl) phthalate (MCPP), mono (6-COOH-2-methylheptyl) phthalate (MCOMHP), monoethyl phthalate (MEP), and mono-isononyl phthalate (mINP) were also assessed.

Phenol analytes included parabens (butyl, ethyl, methyl, and propyl paraben [BuPB, EtPB, MePB, PrPB]), bisphenols (BPA, BPF, BPS), 2,4 and 2,5-dichlorophenol (DCP24, DCP25), benzophenone-3 (BP3), triclocarban (TCC), and triclosan (TCS).

In first trimester urine samples, 17 heavy metals and metalloids were also quantified via isotope chromatography plasma tandem mass spectrometry. These included arsenic (As), barium (Ba), beryllium (Be), cadmium (Cd), chromium (Cr), copper (Cu), mercury (Hg), manganese (Mn), molybdenum (Mo), nickel (Ni), lead (Pb), selenium (Se), tin (Sn), thallium (Tl), uranium (U), tungsten (W), and zinc (Zn).

Concentrations below the limit of detection (LOD) were assigned a value of LOD/√2. Specific gravity of maternal urine samples to indicate urine dilution was measured via a digital handheld device (ATAGO Company, Ltd., Tokyo, Japan). Detection limit and coefficient of variation for the EDC measures has been previously addressed^54^.

### Inflammatory biomarkers

Maternal venous blood samples at two time points (first trimester, and on admission to the hospital prior to delivery), and neonatal umbilical cord blood samples were obtained, divided into aliquots, and stored at −80 degrees Celsius. Cord blood samples were of mixed arterial and venous origin, and obtained and stored within thirty minutes of delivery. Maternal and neonatal plasma samples were then analyzed at the clinical chemistry laboratory at the Michigan Diabetes Research Center (Ann Arbor, MI) for the following inflammatory cytokines: granulocyte macrophage colony-stimulating factor (GM-CSF), interferon (IFN)-γ, monocyte chemotactic protein (MCP)-1, MCP-3, macrophage inflammatory protein (MIP)-1α and MIP-1β, tumor necrosis-factor (TNF)-α, vascular endothelial growth factor (VEGF), interleukin (IL)-1β, IL-6, IL-8, and IL-17α. These 12 cytokines were chosen due to their various roles in immune function. Cytokines were analyzed in duplicates and the average measurement recorded for data analysis. Cytokine levels below the limit of detection (LOD) were replaced by LOD/√2. LOD for individual cytokines was: GM-CSF 0.43 pg/ml, IFN-γ 0.12 pg/ml, MCP-1 1.10 pg/ml, MCP-3 0.24 pg/ml, MIP-1α 1.14 pg/ml, MIP-1β 0.22 pg/ml, TNFα 0.49 pg/ml, VEGF 0.48 pg/ml, IL-1β 0.06 pg/ml, IL-6 0.4 pg/ml, IL-8 0.17 pg/ml, and IL-17α 0.08 pg/ml. Coefficients of variation for individual cytokines were: GM-CSF 9.1%, IFN-γ 6.0%, MCP-1 5.1%, MCP-3 6.3%, MIP-1α 6.4%, MIP-1β 6.5%, TNFα 7.8%, VEGF 18.9%, IL-1β 7.4%, IL-6 10.4%, IL-8 6.5%, and IL-17α 10.5%.

### Birth outcomes

Details on birth outcomes were abstracted from the medical record. Clinical outcomes assessed included estimated gestational age at delivery (calculated by last menstrual period or ultrasound dating), mode of delivery, infant sex, and birth weight. Details on possible confounding variables were provided by subjects at their initial study visit and confirmed in the medical record. Possible confounders included pre-pregnancy body mass index (BMI), maternal age, and history of smoking. Because only two women in our cohort developed gestational hypertension, and only one developed gestational diabetes, pregnancy conditions and complications were not included as confounders.

### Statistical Analysis

To address the issue of measurements falling below the LOD, rank-based methods with ties were applied for correlation and regression analysis that is robust to either fixing truncated measurements at their upper bound or replacing them with LOD/√2.

Spearman correlation coefficients were calculated to determine the association between first trimester exposures and inflammatory cytokine markers at two time points (first trimester and delivery) in mothers and at delivery for newborns. EDC measurements were first adjusted for urine specific gravity and then log-transformed prior to correlation analysis. Each of the 12 inflammatory cytokines was tested for correlation with each of the 41 exposures, and FDR Benjamini–Hochberg (BH) correction was employed to account for multiplicity in the hypothesis testing, using FDR adjusted p-value 0.1 as the cutoff of signal calling. To account for confounding variables, including pre-pregnancy BMI, maternal age, history of smoking, mode of delivery (vaginal versus Cesarean), infant sex, and gestational age at delivery, linear regression was used to examine the association between log-transformed exposures and inflammatory markers. Linear regression was also used to test the association between inflammatory cytokine markers and two birth outcomes, infant gestational age and birthweight. For regression analyses, cytokine values were transformed using inverse normal transformation and birth outcomes were transformed using standard normal transformation. Birthweight z-score was calculated from birthweight in grams, and used as the outcome variable.

To investigate the effect of exposure mixtures on maternal and fetal inflammatory status, we conducted principal component analysis (PCA) on the 41 natural-log-transformed exposure variables. PCA was used in order to simplify the exposure data into fewer dimensions, and to summarize the data into a smaller number of principal components^55^. This resulted in 11 principal components (PC), each representing one type of linear combination of EDC exposure measures, collectively explaining 80% of the total variation in the data. Linear regression was used to evaluate the association between the PCA-weighted EDC exposures with maternal and fetal inflammatory cytokines, adjusted for confounding variables. PCA was also used to investigate the effect of EDC exposure mixtures on infant birth weight and gestational age at delivery. In the models with PC variables, the same inverse normal transformed cytokines and z-scores of birthweight were used, and p < 0.05 was considered significant. Statistical analysis was completed in R Software, version 3.0.

In addition to the cross-sectional data analysis of inflammatory cytokine measurements at each time point, we conducted a longitudinal analysis using a Linear Mixed Model (LMM) with random intercepts to account for temporal association between repeatedly measured concentrations of maternal cytokine levels in first trimester and at term. The LMM model, with interaction terms between time and PCA-weighted EDC exposures, analyzed both main effects of the PC-weighted EDC exposure and time, and more importantly how the association effect between the exposures and cytokines may have shifted from baseline to term.

### Ethics approval and consent to participate

This study was approved by the University of Michigan IRB Committee.

## Results

Subjects in the current analysis represent 56 mother-infant dyads in the MMIP cohort. Sample characteristics and infant birth outcomes are outlined in Table 1. First trimester urinary concentrations of metals, phenol and phthalate metabolites for the 56 women in this cohort have been previously detailed^54^. In general, women displayed an average of 30 detected EDC exposures during the first trimester, including a mean of 12.8 metals, 10.5 phthalate metabolites, and 7.7 phenols^54^.

**Table 1:** Subject characteristics and birth outcomes (n = 56).

	N	%	Mean	Standard Deviation	Range
Maternal age (years)			31.9	3.7	26–40
Maternal pre-pregnancy weight (kg)			70.1	15.8	51–135
Gravidity			2.11	1.02	1–5
Parity			1.29	1.06	0–4
Married	56	100			
White Race	56	100			
Current Smoker	0	0			
Former Smoker	6	10.7			
Education					
High school	4	7.1			
Some College	34	60.7			
College Graduate	18	32.1			
Income ($USD)					
<49,000	8	14.3			
50,000–100,000	22	39.3			
>100,000	26	46.4			
Gestational age at delivery (weeks)			39.7	1.02	37.6–41.7
Infant birth weight (g)			3552	396.6	2805–4685
Mode of delivery					
Vaginal	37	66			
Cesarean	19	34			
Infant sex					
Female	29	52			
Male	27	48			

**Figure 1:**
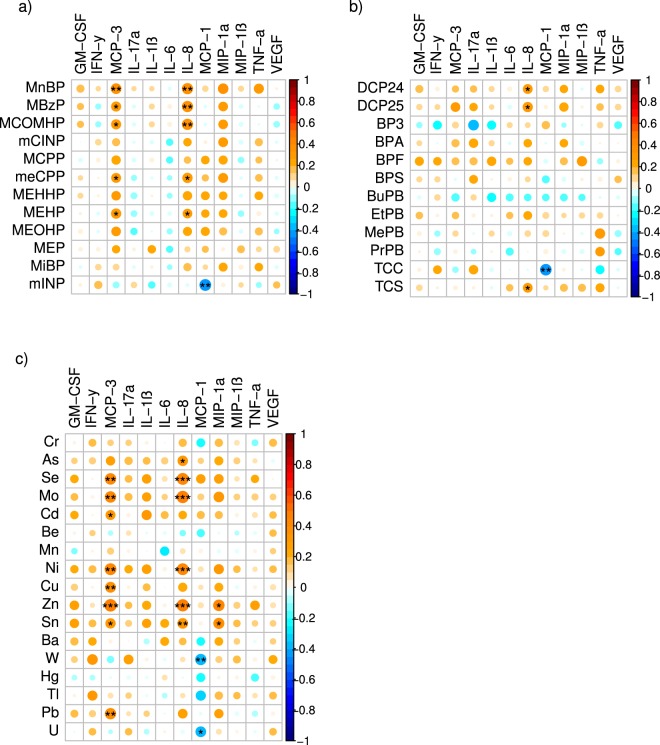
Spearman correlations between early pregnancy EDC exposures and first trimester inflammatory cytokines. Using the color spectrum, the orange color indicates a positive correlation, while the blue color indicates a negative correlation for cytokines with (**a**) phthalates, (**b**) phenols, and (**c**) metals. Circle size reflects the size of the correlation, with larger circles having correlations closer to 1 or −1. Shade of orange or blue also reflects the strength of the correlation coefficient. Significance is noted as follows: ^•••^p < 0.01, ^••^p < 0.05, ^•^p < 0.10.

**Figure 2:**
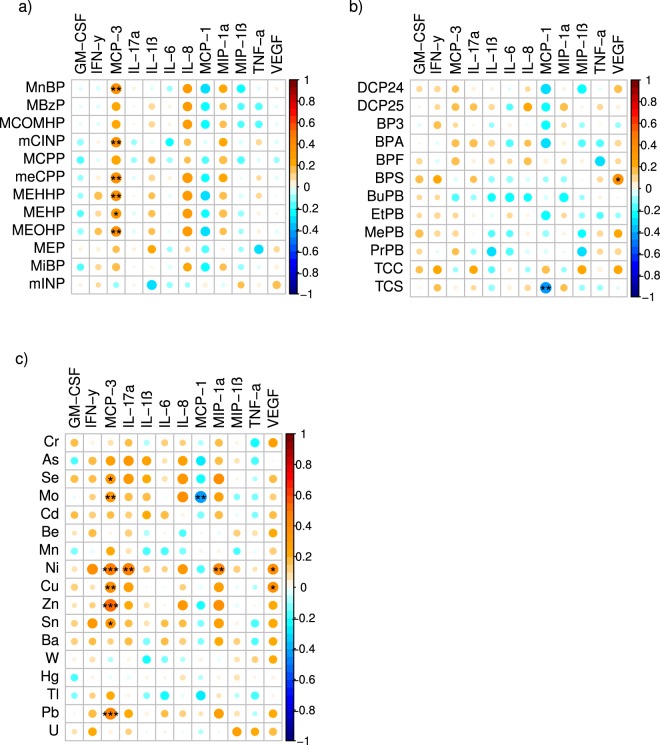
Spearman correlations between early pregnancy EDC exposures and term inflammatory cytokines. Using the color spectrum, the orange color indicates a positive correlation, while the blue color indicates a negative correlation for cytokines with (**a**) phthalates, (**b**) phenols, and (**c**) metals. Circle size reflects the size of the correlation, with larger circles having correlations closer to 1 or −1. Shade of orange or blue also reflects the strength of the correlation coefficient. Significance is noted as follows: ^•••^p < 0.01, ^••^p < 0.05, ^•^p < 0.10.

**Figure 3:**
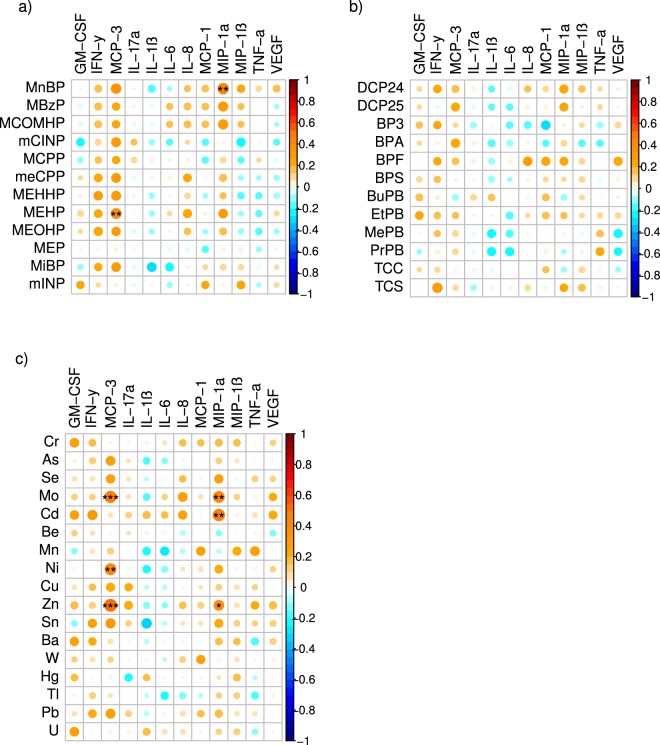
Spearman correlations between early pregnancy EDC exposures and neonatal cord blood inflammatory cytokines. Using the color spectrum, the orange color indicates a positive correlation, while the blue color indicates a negative correlation for cytokines with (**a**) phthalates, (**b**) phenols, and (**c**) metals. Circle size reflects the size of the correlation, with larger circles having correlations closer to 1 or −1. Shade of orange or blue also reflects the strength of the correlation coefficient. Significance is noted as follows: ^•••^p < 0.01, ^••^p < 0.05, ^•^p < 0.10.

First trimester measures of several individual EDCs, particularly metals and phthalates, were independently associated with maternal first trimester and term inflammatory markers using Spearman correlation coefficients (Figs 1 and 2). Positive correlations were frequently observed between individual EDC exposures and maternal levels of MCP-3, IL-8, and MIP-1α. There were fewer observed correlations between individual EDC exposures and inflammatory markers in cord blood (Fig. 3).

When adjusted for confounders using linear regression, individual EDCs were significantly associated with maternal IL-6 in the first trimester, and maternal MCP-1 and IL-17 at delivery. Table 2 demonstrates the directionality of these associations. The FDR Benjamini-Hochberg (BH) correction was used to control for false discoveries related to multiple comparisons, and it is standard to use a false discovery rate of 0.10. Thus, we used BH adjusted p-value < 0.10.

**Table 2:** EDC associations with maternal cytokine levels in early and late pregnancy.

Pregnancy Time Point	EDC	Category	Cytokine	Effect Size (*SE*)	BH-adjusted p-value
First trimester	BuPB	Phenol	IL-6	−0.319 (*0.106*)	0.097
First trimester	Ba	Metal	IL-6	0.450 (*0.150*)	0.097
Term	Mo	Metal	MCP-1	−0.859 (*0.217*)	0.012
Term	BPA	Phenol	MCP-1	0.816 (*0.214*)	0.019
Term	TCS	Phenol	MCP-1	−0.509 (*0.158*)	0.051
Term	Ni	Metal	IL-17α	−0.265 (*0.087*)	0.055
Term	Se	Metal	IL-17α	0.931 (*0.310*)	0.066
Term	As	Metal	IL-17α	0.433 (*0.145*)	0.066
Term	Mo	Metal	IL-17α	0.612 (*0.219*)	0.075
Term	Cu	Metal	IL-17α	0.649 (*0.237*)	0.075

Table 2 displays linear regression results for the association between EDC and maternal cytokine levels, after adjusting for confounding variables including urine dilution, maternal pre-pregnancy BMI, maternal age, history of smoking, mode of delivery, infant sex, and gestational age at delivery. Only statistically significant associations at a Benjamini-Hochberg adjusted p-value < 0.10 are displayed here. No significant associations with cord blood cytokines were observed.

Several positive correlations between inflammatory markers within each time point (first trimester, term, and post-delivery) were identified (Supplemental Fig. 2). For instance, in the first trimester, examples of significant, positive correlations include IFN-γ with IL-17, MCP-3 with IL-8, and MIP-1α with IL-8. In addition, several positive associations were observed between inflammatory marker levels in first trimester, at term, and in cord blood, with infant gestational age and birth weight at delivery (Table 3). For instance, first trimester and term levels of IL-8 were positively associated with birth weight, and cord blood VEGF was positively associated with gestational age.

**Table 3:** Cytokine associations with birth outcomes.

Pregnancy Time Point	Cytokine	Birth Outcome	Effect Size (*SE*)	P-value
First trimester	MIP-1α	Birthweight	0.198 (*0.094*)	0.043
First trimester	IL-8	Birthweight	0.184 (*0.093*)	0.056
First trimester	TNF-α	Birthweight	0.185 (*0.102*)	0.077
First trimester	IL-6	Gestational age	1.907 (*1.128*)	0.098
Term	IL-8	Birthweight	0.248 (*0.097*)	0.014
Term	IL-1β	Gestational age	1.859 (*1.087*)	0.094
Cord blood	MIP-1α	Birthweight	0.205 (*0.094*)	0.034
Cord blood	IL-1	Birthweight	0.174 (*0.101*)	0.094
Cord blood	VEGF	Gestational age	2.182 (*1.142*)	0.063

Table 3 displays linear regression results for the association between inflammatory cytokines and birth outcomes. Adjustments were made for confounding variables including urine dilution, maternal pre-pregnancy BMI, maternal age, history of smoking, mode of delivery, and infant sex. Only associations considered significant with p-value < 0.10 are displayed here.

Tables 4–6 demonstrate each of the specific PC groupings which were significantly associated (p < 0.05) with maternal first trimester, maternal term, and delivery cord blood inflammation, respectively.

**Figure 4:**
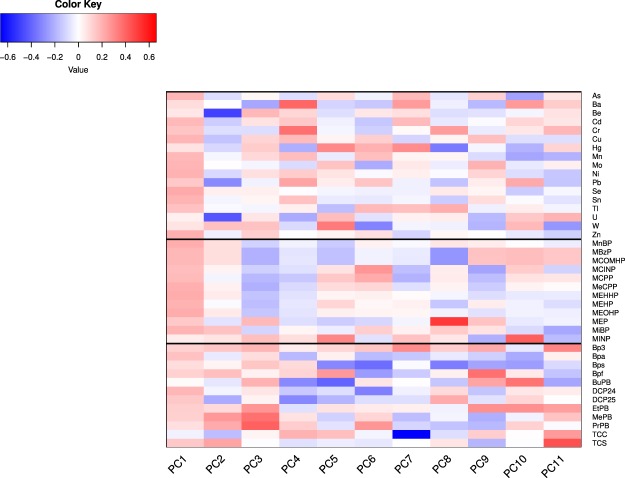
Principal component loading coefficients. This color heat map shows the loading coefficients of the exposures for each of the principal components (PC), demonstrating the relative contribution of each EDC to a specific PC group. Exposures that have a higher positive weight are darker red, whereas exposures that are negatively weighted are blue. Tables 4–6 demonstrate each of the specific PC groupings which were significantly associated with maternal first trimester, maternal term, and delivery cord blood inflammation, respectively. By analyzing the components of a significant PC, one can interpret the association results. For example, in Table 4, PC1 is shown to have positive association with IL-8 and IFN- γ. From Fig. 4, PC1 is positively weighted towards metals and phthalates (red color), suggesting that higher metal and phthalate levels may be positively associated with first trimester IL-8 and IFN-γ.

Ten of eleven PC groupings demonstrated statistically significant associations with inflammatory cytokines. Tables 4–6 demonstrate each of the specific PC groupings which were associated with maternal first trimester, maternal term, and delivery cord blood inflammation, respectively. For interpretation of these PCA results, the color heat map (Fig. 4) demonstrates the relative contribution of each EDC to a specific PC group. For instance, in PC1, metals and phthalates are positively weighted (red color), suggesting that higher metal and phthalate levels may be positively associated with first trimester IL-8 and IFN-γ (Table 4). Similarly, phenols are predominantly negatively weighted in PC6 (blue color), and in the first trimester, this PC grouping demonstrated negative associations with IL-1β, IL-17α, VEGF, and GM-CSF levels. Because of the similar trend in directionality, this suggests that phenols may be positively correlated with these individual cytokines (Table 4). Using the same 11 PC variables, there were no significant associations between EDC mixtures with infant birth weight or gestational age at delivery.

**Table 4:** Association of EDC mixtures with first trimester cytokines.

PC variable	Maternal cytokines (first trimester)	
	Positive Association	Negative Association
PC 1	IL-8, IFNγ	
PC 4	IL-6, IL-8, GM-CSF	
PC 5	IL-8	
PC 6		IL-1β, IL-17α, VEGF, GM-CSF
PC 11	IL-6	IFNγ

**Table 5:** Association of EDC mixtures with maternal cytokines at term.

PC variable	Maternal cytokines (term)	
	Positive Association	Negative Association
PC 1	IL-17α, IFNγ	MCP-1
PC 2		IL-1β
PC 4	VEGF, IL-6, IL-17α, MCP-1, GM-CSF, MIP-1α	TNFα
PC 5	IL-8, MIP-1α, MCP-3	
PC 9	IL-17α, IL-1β	
PC 10		IL-17α
PC 11	IL-6	MCP-1

**Table 6:** Association of EDC mixtures with cord blood cytokines.

PC variable	Neonatal cytokines (cord blood)	
	Positive Association	Negative Association
PC 1	MCP-3	
PC 5	MCP-3	
PC 7	VEGF, MIP-1α, GM-CSF	
PC 8		IL-8, MCP-1
PC 10	MIP-1α, IL-8	
PC 11	IL-6, IL-1β	

In the Linear Mixed Model (LMM) longitudinal analysis, there were statistically significant interactions between time of maternal inflammatory measurement and seven of the PC groupings. These interactions indicate an altered association from baseline to term, which are not captured by the cross-sectional analyses conducted by linear regression. Table 7 demonstrates these seven groupings and the related (p < 0.05) inflammatory cytokines. The positive values indicate an increased association at term from baseline. The main effects in the LMM model also demonstrated statistically significant associations between the PC groupings and the maternal baseline inflammatory levels. As expected, these associations were similar to the cross-sectional linear regression analysis on maternal first trimester cytokines (results not shown).

**Table 7:** Change in association of EDC mixtures with maternal cytokines from first trimester to term.

PC variable	Maternal cytokines (difference from baseline to term)	
	Positive Association	Negative Association
PC 1		MCP-1, TNFα, MIP-1β
PC 2		IL-17α, MIP-1β
PC 4	VEGF	IL-1
PC 6	IL-1, MIP-1α, MIP-1β	
PC 9	IL-1	
PC 10		MIP-1α, IL-8
PC 11	IFNγ	

Finally, we performed comparative analyses between several subgroups, using mean concentrations (95% CI) of EDC measures and inflammatory markers: maternal obese vs. non-obese, primiparous vs. multiparous, and neonatal sex (male vs. female). However, no significant differences were identified (data not shown).

## Discussion

Women in this Michigan-based birth cohort demonstrated detectable exposure to an average of 30 chemicals and metals, out of 41 possible that were measured, in the first trimester. These results are comparable to population studies such as NHANES, which demonstrated a median detectable exposure of pregnant women to 50 chemical analytes, out of 71 possible exposures^15^. Pregnant women in the MMIP cohort demonstrated lower urinary levels of many exposures, such as Hg (geometric mean 0.07 ug/L [95% CI 0.06–0.09] in MMIP, vs geometric mean 0.45 ug/L in a San Francisco-based pregnancy cohort) and BPA (geometric mean 0.71 [0.52–0.98] in MMIP, vs 50^th^ percentile 2.7 ug/L in NHANES)^15,56^. However, some exposure measures appeared to be greater in MMIP subjects, such as TCS (geometric mean 14.5 ug/L [8.28–25.40] vs 50^th^% percentile 8.2 ug/L in NHANES). These wide ranges of values, both within and between cohorts, may reflect differential exposure due to geography, socioeconomic status, occupation, and other environmental factors.

Furthermore, in this sample of pregnant women and their full-term newborns, inflammatory marker levels in early gestation and the peripartum period were differentially associated with (1) individual exposures and (2) exposure mixtures. These findings suggest that not only do these environmental exposures alter the maternal-fetal inflammasome throughout pregnancy, but that the combination of exposures is of particular importance. Furthermore, several positive associations between individual inflammatory measures and two birth outcomes, infant birth weight and gestational age, were also evident.

Under normal conditions, maternal inflammation is thought to increase throughout pregnancy. One proposed rationale is that systemic inflammation induces maternal insulin resistance, which increases the availability of glucose for fetal growth^57,58^. For the 12 inflammatory markers measured in our study, our subjects (excluding outliers) demonstrated similar cytokine levels in the first trimester and at term (Supplemental Fig. 1). These findings differ from a recently published study of 221 pregnant women, where serum levels of IL-8 and TNF-α were significantly higher in the third trimester compared to the first trimester^59^. Notably, that cohort had a larger sample size with a 13.9% preterm birth rate, which contrasts with our smaller cohort of full-term pregnancies. The different patterns of IL-8 and TNF-α levels may be explained by these population differences. Furthermore, inflammatory cytokine measures were only performed on plasma samples in our study. It is possible that cytokine profiles over time would have demonstrated more pronounced changes if these measures were also performed in amniotic fluid. Amniotic fluid cytokines are thought to have an important role in parturition, including rupture of fetal membranes, cervical dilation, and uterine contractility^60^. For instance, one study demonstrated that in healthy pregnant women, amniotic fluid samples had higher levels of IL-6, IL-8, and MCP-1 than matched blood samples, while levels of TNF-α, IFN-γ, and VEGF were higher in serum^61^. Although amniotic fluid cytokines may more accurately reflect fetal-placental inflammation, the invasive nature of amniocentesis limited the availability of these samples in our study.

Our findings also suggest that maternal exposure to EDCs may contribute to perturbations in antepartum and peripartum cytokine levels in not only the pregnant mother, but also the newborn. A unique aspect of our study is that maternal inflammatory markers were related to EDCs measured in early pregnancy (8–14 weeks’ gestation), a critical window of fetal and placental development. In relation to individual exposures, increased first trimester exposure to metals and phthalates was found to be positively associated with MCP-3 and IL-8 levels, while increased exposure to metals was negatively associated with MCP-1. However, when adjusted for confounders (urine dilution, maternal pre-pregnancy BMI, maternal age, history of smoking, mode of delivery, infant sex, and gestational age at delivery), first trimester IL-6 was the only cytokine with a significant association to environmental EDCs (positively correlated with Ba, and negatively correlated with BuPB). One published study involving a Puerto Rican pregnancy cohort^45^ found an association between TCS exposure and increased IL-6 levels at two time points in the second trimester (16–20 weeks, and 24–28 weeks) and a similar trend between the phthalate metabolite DEHP with IL-6^62^. In contrast, in our cohort of Caucasian women, neither TCS nor DEHP metabolites were found to be individually associated with IL-6. These differences may relate to differences in the timing of EDC and inflammatory measures, in addition to ethnic differences. Another pregnancy cohort in Boston demonstrated a link between maternal BPA and IL-6 levels at various points in pregnancy, including the first trimester^63^. This finding was not observed in our MMIP population, possibly due to differences in patient demographics, regional exposures, and/or statistical power.

At term, just prior to delivery, we found that maternal MCP-3 maintained positive relationships with first trimester measures of metals and phthalates. Interestingly, the correlation between MCP-3 and first trimester metal exposures was also demonstrated in umbilical cord blood, although this correlation disappeared after adjusting for confounding variables. MCP-3 attracts monocytes and macrophages but its specific role in pregnancy has not been defined^64^. Its relevance to maternal-fetal outcomes warrants further investigation.

There is limited data on the relationship between EDC exposure with cord blood markers, despite the fact that cord blood samples are relatively easy to obtain. Measures of cord blood exposures may be an effective proxy for maternal exposures during pregnancy, as chemicals in maternal blood are often also detected in cord blood^65^. To our knowledge, ours is the first study to directly compare neonatal cord blood inflammation with early trimester maternal EDC measures. Assessing maternal exposure in early pregnancy, and not at the time of delivery, allows investigation of the maternal exposome during important periods in fetal development, which is a unique feature of our study.

Use of principal component analysis (PCA) identified several notable associations between EDC mixtures and maternal/neonatal cytokine levels. These findings contribute to a growing body of literature on the impact of chemical mixtures on human health^66,67^. Notably, comparison of individual EDCs to various groupings of EDC mixtures demonstrated different associations with cytokine levels. The principal component (PC) group 11 in our dataset provides an excellent example of this. As evidenced in Tables 4–6, PC 11 is weighted positively with TCS and BP3, and this particular chemical grouping is positively associated with IL-6 in maternal baseline, term, and fetal cord blood. However, when assessing TCS or BP3 as isolated exposures, no relationships with IL-6 were demonstrated. These differences reinforce the principle that EDCs may act in summation, negation, or synergy with one another, and that the chemical mixtures to which humans are exposed are critically important in assessing environmental exposure risk^68^. In addition, the observed longitudinal changes of inflammatory biomarkers from first trimester to term, in relation to EDC mixtures, suggest that EDC exposures may alter the physiologic cytokine shifts which occur throughout pregnancy. However, the exact contribution of these combined exposures to longitudinal cytokine levels, and the clinical implications of these findings, remain unknown at this time.

In practice, there are methodologic challenges to studying mixture interactions and their physiologic effects^66,67^. Some chemical combinations are more prominent than others depending upon socioeconomic, occupational, dietary, behavioral, and geographic factors of the study population^69,70^. More research is needed to identify the most prevalent exposure mixtures among specific populations, and their cumulative impact on physiologic processes^66^. Although the EDC exposure assessment undertaken in this study was comprehensive, with urinary measures of 41 chemicals, in reality humans are exposed to innumerable chemicals via various routes of administration including oral, inhaled, dermal, and ingested. The complex nature of these “real life” exposures, and the challenges of quantifying them, is demonstrated in assessing environmental exposure risks in the agriculture, oil and gas industries^71,72^. In addition, EDCs may modulate downstream molecular and cellular pathways in a dose-independent, non-monotonic, sex-specific manner, and may be influenced by other factors such as genetics, nutrition and stress^2^, which may be difficult to account for.

While several epidemiologic studies demonstrate an association between EDC burden and low birth weight in humans^73–76^, some studies demonstrate no association^25,77,78^. In our 56 subjects, we only observed associations between birth weight and two exposures, MCPP (positive) and BPS (negative)^54^. However, associations between inflammatory cytokine levels and birth outcomes were evident. Our findings revealed higher levels of first trimester MIP-1α, IL-8, and TNF-α to be associated with an increase in infant birth weight, while first trimester IL-6 was found to be associated with an increase in gestational age. In contrast, no significant associations were found between the inflammatory milieu at the time of delivery with birth outcomes. These observations have important translational value. Aberrant inflammation in pregnancy has been associated with a spectrum of adverse outcomes related to implantation, placentation, and fetal growth, including implantation failure, recurrent pregnancy loss, preeclampsia, intrauterine growth restriction, and preterm birth^47,50,79–83^. A link with inflammation is particularly well-established in the preterm population^84–86^. In our cohort, all newborns were delivered at term, which may explain why an association between gestational age and inflammation at delivery was not observed. Further, all newborns in our cohort were of normal birth weight. Although in the original DOHaD hypothesis^87^, low birth weight was the primary risk factor for adult-onset disease, there is increasing evidence that developmental programming may occur in a variety of pathways, including inflammatory, endocrine, epigenetic, nutritional, oxidative stress, and/or placental^88–91^. Our findings of an altered inflammatory milieu in utero may still confer long-term health programming effects on offspring, even in the setting of normal birth weight.

To our knowledge, this is the first study to support an association between EDC mixtures and alterations in the maternal and neonatal inflammatory environment. Similar to other studies^16,92^, we demonstrate that reproductive age women are widely exposed to multiple classes of environmental chemicals. Because gestational EDC exposures may have long-term health implications for adult offspring through developmental programming, potentially due to inflammatory mechanisms, this particular area of research has significant clinical and public health implications for women of childbearing age^69,70,93^.

Strengths of our study include our comprehensive assessment of the association between 41 first trimester exposures, with 12 inflammatory cytokines in early pregnancy and the peripartum period. Recruitment of pregnant women at their first prenatal appointment allowed assessment of the maternal inflammasome in the first trimester of pregnancy, when fetal development is particularly sensitive to insults. Our study is limited by a small sample size and a non-diverse patient population. However, our subject homogeneity does limit the potential for additional confounding variables including race, education, and socioeconomic status. As previously mentioned, EDCs may exert their effects in a sex-specific manner^94^, but due to small sample size, male and female newborns were not analyzed separately. Limitations of our study design include the fact that we did not account for hemodilution or hemoconcentration of plasma samples. Furthermore, EDC exposures were only measured at a single time point in pregnancy. Exposure measures may vary throughout pregnancy, and repeated measures of EDC levels over time may better represent average prenatal exposure^95,96^. By only measuring EDCs in the first trimester, we were unable to assess the impact of second or third trimester exposures on inflammatory processes. Other possible confounders, which were not addressed by our statistical design, include pre-pregnancy maternal comorbidities, maternal weight gain in pregnancy, parity, medication use, vitamin or supplement intake during pregnancy, and whether labor was initiated spontaneously or via induction. Finally, although our pilot study is unable to assess mechanisms or establish causality, our findings highlight the potential association of circulating inflammatory markers in pregnancy with EDC mixtures, which warrants additional research.

## Conclusions

In conclusion, inflammatory cytokine levels throughout pregnancy and at delivery may be explained by unique mixtures of first trimester environmental toxicant exposures. More research is needed to validate these findings on a larger scale and to assess their impact on a wider range of pregnancy and birth outcomes.

## Supplementary information

Supplemental Figures/Tables

## Data Availability

The data analyzed during the current study are not publicly available, but can be made available from the corresponding author on reasonable request.
