# Supplementary material for: Early pregnancy exposure to endocrine disrupting chemical mixtures are associated with inflammatory changes in maternal and neonatal circulation
Source: Sci Rep. 2019 Apr 1;9:5422. doi: 10.1038/s41598-019-41134-z (PMC6443771; doi:10.1038/s41598-019-41134-z)
Supplement: Supplementary file 1 — Supplemental Figures/Tables [file 41598_2019_41134_MOESM1_ESM.pdf]

SUPPLEMENTARY MATERIAL

Early pregnancy exposure to endocrine disrupting chemical mixtures are associated with inflammatory changes in maternal and neonatal circulation

Angela S Kelley[1*], Margaret Banker[2*], Jaclyn M Goodrich[3], Dana C Dolinoy [3,4], Charles Burant[5], Steven E Domino[1], Yolanda R Smith[1], Peter XK Song[2], Vasantha Padmanabhan[1,3,6]

*Co-first authors

[1] Department of Obstetrics and Gynecology, University of Michigan, L4001 Women's Hospital, 1500 East Medical Center Drive, Ann Arbor, Michigan, 48109, USA. Email: asimmen@med.umich.edu (AK), sedomino@med.umich.edu (SD), ysmith@med.umich.edu (YS)

[2] Department of Biostatistics, University of Michigan School of Public Health, 1415 Washington Heights, Ann Arbor, Michigan, 48109, USA. Email: mbanker@umich.edu (MB), pxsong@umich.edu (PS)

[3] Department of Environmental Health Sciences, University of Michigan School of Public Health, 1415 Washington Heights, Ann Arbor, Michigan, 48109, USA. Email: gaydojac@umich.edu (JG), ddolinoy@umich.edu (DD)

[4] Department of Nutritional Sciences, University of Michigan School of Public Health, 1415 Washington Heights, Ann Arbor, Michigan, 48109, USA

[5] Department of Internal Medicine, University of Michigan, 24 Frank Lloyd Wright Drive, Ann Arbor, Michigan, 48105, USA. Email: burantc@med.umich.edu (CB)

[6] Department of Pediatrics, University of Michigan, 7510 MSRB 1, 1500 W. Medical Center Dr, Ann Arbor, MI 48109, USA. Email: vasantha@umich.edu (VP)

Supplemental Figure 1. Longitudinal differences between maternal baseline and term cytokine levels.

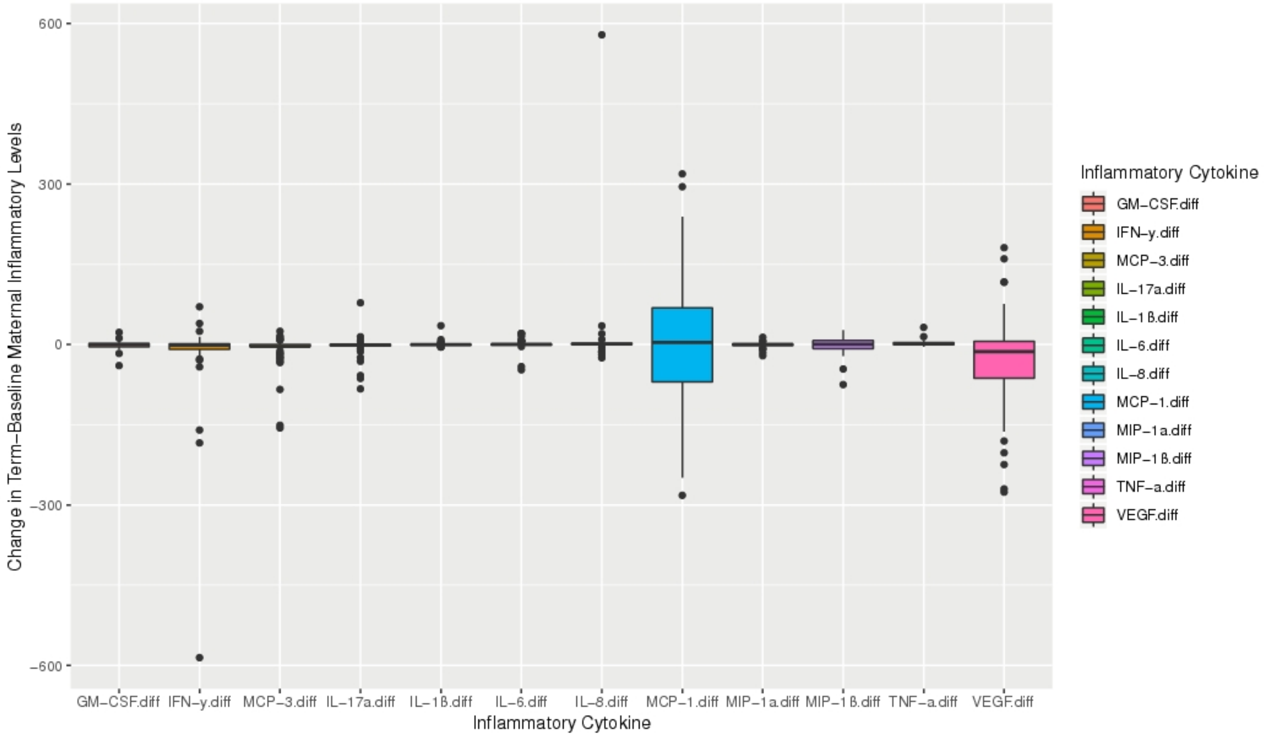

Supplemental Figure 2. Spearman correlations between cytokines measured in first trimester, at term, and in cord blood.

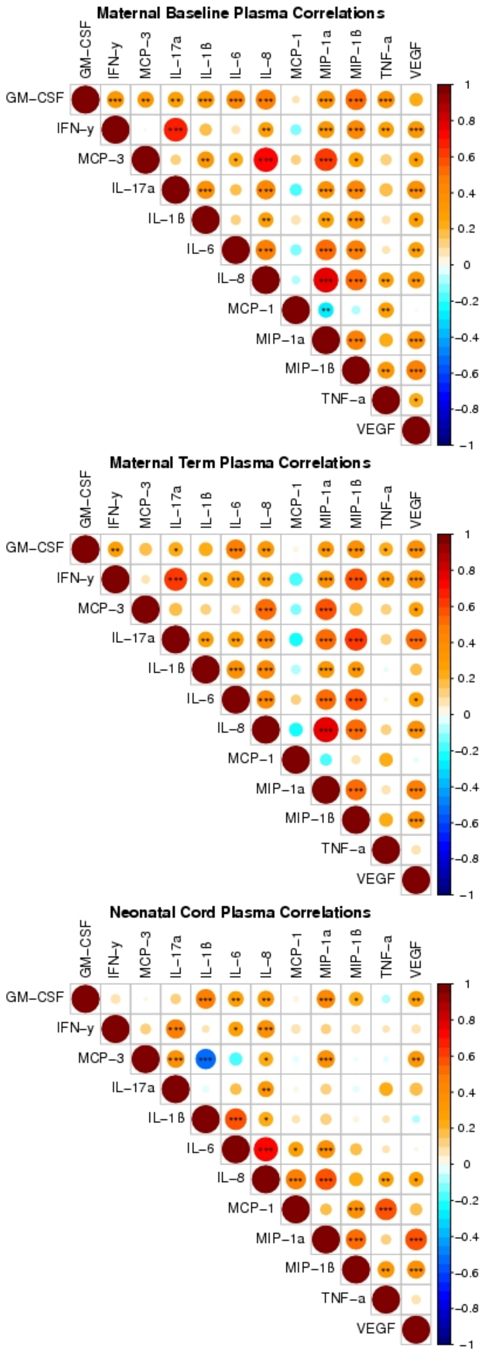

Supplemental Table 1: Urinary measures of first trimester exposures in MMIP (n = 56*)

Urinary Analyte	Geometric Mean, *u*g/L	95% CI, *u*g/L
As	4.30	(3.07, 6.00)
Ba	5.00	(3.88, 6.44)
Be	0.04	(0.03, 0.04)
Cd	0.08	(0.07, 0.11)
Cr	0.69	(0.60, 0.80)
Cu	8.10	(6.64, 9.89)
Hg	0.07	(0.06, 0.09)
Mn	1.44	(1.20, 1.73)
Mo	49.20	(38.31, 63.19)
Ni	3.77	(3.06, 4.64)
Pb	0.22	(0.17, 0.28)
Se	41.63	(33.23, 52,15)
Sn	0.31	(0.23, 0.43)
Tl	0.16	(0.13, 0.20)
U	0.01	(0.01, 0.01)
W	0.16	(0.14, 0.17)
Zn	169.87	(129.09, 223.54)
MnBP	6.87	(5.11, 9.23)
MBzP	4.20	(2.91, 6.06)
MCOMHP	4.18	(2.89, 6.04)
mCINP	1.29	(0.98, 1.71)
MCPP	1.38	(0.90, 2.11)
MeCPP	5.14	(3.79, 6.97)
MEHHP	5.30	(3.92, 7.16)
MEHP	1.93	(1.51, 2.47)
MEOHP	2.76	(2.03, 3.76)
MEP	18.04	(12.20, 26.67)
MiBP	3.34	(2.37, 4.70)
mINP	0.36	(0.34, 0.39)
DCP24	0.40	(0.29, 0.54)
DCP25	0.42	(0.28, 0.63)
BP3	58.35	(34.52, 98.61)
BPA	0.71	(0.52, 0.98)
BPF	0.26	(0.20, 0.36)
BPS	0.21	(0.18, 0.26)
BuPB	0.27	(0.20, 0.37)
EtPB	1.07	(0.57, 2.00)

MePB	53.08	(32.20, 87.49)
PrPB	8.21	(4.67, 14.42)
TCC	0.17	(0.14, 0.21)
TCS	14.50	(8.28, 25.40)

*For metals, n=55.

Supplemental Table 2. Inflammatory cytokine values measured at baseline (first trimester), term, and in cord blood.

	Baseline	Term	Umbilical Cord
Cytokine	Mean (SD) (pg/mL)	Mean (SD) (pg/mL)	Mean (SD) (pg/mL)
GM-CSF	7.75 (10.57)	7.02 (10.48)	2.30 (3.12)
IFN-*y*	32.47 (89.56)	14.64 (25.85)	3.39 (11.27)
MCP-3	66.13 (126.80)	55.41 (101.56)	40.01 (90.19)
IL-17α	16.16 (36.93)	12.05 (31.89)	0.67 (0.67)
IL-1	2.10 (1.83)	2.46 (5.05)	1.03 (1.53)
IL-6	6.60 (17.95)	7.00 (13.76)	41.78 (136.42)
IL-8	12.10 (20.21)	22.01 (79.73)	25.62 (52.97)
MCP-1	260.41 (90.56)	263.80 (93.37)	640.22 (527.89)
MIP-1α	8.82 (9.89)	8.16 (8.41)	7.20 (8.20)
MIP-1β	24.03 (19.10)	22.25 (17.58)	35.18 (23.83)
TNF-α	6.05 (2.88)	8.26 (5.61)	17.07 (6.12)
VEGF	114.25 (161.61)	82.34 (129.69)	69.06 (152.79)

<u>Supplemental Data for Tables 4-7</u>

Supplemental Dataset - Table 4

**Model: Linear Regression - Maternal Baseline**				
Inflammatory Cytokine	*PC Grouping*	*Estimate*	*Standard Error*	*p-value*
IL-6	PC4	0.34	0.09	0.00
IL-1	PC6	(0.35)	0.11	0.00
IL-17α	PC6	(0.33)	0.11	0.01
IL-6	PC11	0.36	0.12	0.01
IL-8	PC1	0.15	0.05	0.01
IFN-γ	PC11	(0.44)	0.16	0.01
IL-8	PC4	0.28	0.11	0.02
VEGF	PC6	(0.28)	0.12	0.02
IFN-γ	PC1	0.14	0.06	0.03
IL-8	PC5	0.24	0.11	0.04
GM-CSF	PC6	(0.24)	0.11	0.04
GM-CSF	PC4	0.23	0.11	0.04

Supplemental Dataset - Table 5

**Model: Linear Regression - Maternal Term**				
Inflammatory Cytokine	*PC Grouping*	*Estimate*	*Standard Error*	*p-value*
IL-17α	PC1	0.17	0.05	0.00
VEGF	PC4	0.34	0.11	0.00
IL-6	PC11	0.43	0.14	0.01
MCP-1	PC1	(0.17)	0.05	0.01
IL-17α	PC9	0.37	0.12	0.01
IL-17α	PC10	(0.33)	0.12	0.01
MCP-1	PC11	(0.38)	0.14	0.01
IL-1	PC9	0.36	0.13	0.01
IL-1	PC2	(0.25)	0.10	0.02
IL-6	PC4	0.26	0.11	0.02
IL-17α	PC4	0.23	0.10	0.02
IFN-γ	PC1	0.13	0.06	0.03
MCP-1	PC4	0.25	0.11	0.03
IL-8	PC5	0.31	0.13	0.03
GM-CSF	PC4	0.27	0.13	0.04
MIP-1α	PC5	0.26	0.13	0.05
TNF-α	PC4	(0.22)	0.11	0.05
MIP-1α	PC4	0.25	0.12	0.05
MCP-3	PC5	0.25	0.12	0.05

Supplemental Dataset - Table 6

**Model: Linear Regression – Neonatal Cord**				
Inflammatory Cytokine	*PC Grouping*	*Estimate*	*Standard Error*	*p-value*
MCP-3	PC5	0.36	0.12	0.00
IL-8	PC8	(0.43)	0.14	0.01
IL-6	PC11	0.36	0.14	0.01
MCP-3	PC1	0.14	0.06	0.02
MCP-1	PC8	(0.34)	0.15	0.03
VEGF	PC7	0.33	0.14	0.03
MIP-1α	PC10	0.31	0.14	0.03
IL-1	PC11	0.31	0.14	0.04
MIP-1α	PC7	0.29	0.13	0.04
IL-8	PC10	0.29	0.14	0.05
GM-CSF	PC7	0.30	0.14	0.05

Supplemental Dataset - Table 7

**Model: LMM - Main Effects**				
Inflammatory Cytokine	*PC Grouping*	*Estimate*	*Standard Error*	*p-value*
IL-17α	PC6	(0.33)	0.10	0.00
IL-1	PC6	(0.32)	0.11	0.01
IL-6	PC4	0.30	0.10	0.01
IFN-γ	PC11	(0.43)	0.15	0.01
IL-6	PC11	0.37	0.13	0.01
VEGF	PC6	(0.29)	0.11	0.02
IFN-γ	PC1	0.13	0.05	0.02
IL-17α	PC1	0.11	0.05	0.02
IL-8	PC1	0.13	0.06	0.03
TNF-α	PC1	0.12	0.05	0.04
GM-CSF	PC4	0.24	0.12	0.05
IL-8	PC4	0.25	0.12	0.05
GM-CSF	PC6	(0.24)	0.12	0.06
VEGF	PC4*time	0.22	0.10	0.04
IL-17α	PC2*time	(0.16)	0.08	0.05
**Model: LMM - Interaction Effect**				
Inflammatory Cytokine	*PC Grouping*	*Estimate*	*Standard Error*	*p-value*
MCP-1	PC1*time	(0.15)	0.04	0.00
TNF-α	PC1*time	(0.11)	0.03	0.00
IL-1	PC4*time	(0.28)	0.09	0.00
IFN-γ	PC11*time	0.50	0.16	0.00
MIP-1β	PC2*time	(0.19)	0.07	0.01
MIP-1β	PC1*time	(0.07)	0.03	0.01
IL-1	PC6*time	0.25	0.09	0.01
MIP-1α	PC10*time	(0.18)	0.07	0.02
MIP-1α	PC6*time	0.14	0.06	0.02
MIP-1β	PC6*time	0.21	0.09	0.03
IL-8	PC10*time	(0.26)	0.11	0.03
IL-1	PC9*time	0.24	0.11	0.04
VEGF	PC4*time	0.22	0.10	0.04
IL-17α	PC2*time	(0.16)	0.08	0.05
